# Assessment of Depression, Anxiety, and Quality of Life in Patients With Psoriasis Using the Dermatology Life Quality Index (DLQI) and Depression Anxiety Stress Scale-21 (DASS-21): A Cross-Sectional Study From Southern India

**DOI:** 10.7759/cureus.109326

**Published:** 2026-05-21

**Authors:** Naveen Manohar, Gajanan A Pise, Shruthi S Prasad, Shilpa V Dastikop, Abhineetha Hosthota, Malteshgouda N Patil

**Affiliations:** 1 Dermatology, The Oxford Medical College, Hospital and Research Centre, Bengaluru, IND; 2 Dermatology, Belagavi Institute of Medical Sciences, Belagavi, IND; 3 Dermatology, Indira Medical College and Hospitals, Pandur, IND

**Keywords:** anxiety, dass-21, depression, dlqi, psoriasis vulgaris, psychodermatology, quality-of-life

## Abstract

Introduction

Psoriasis is a chronic inflammatory skin disorder associated with significant psychosocial morbidity. Despite its visible nature and impact on daily functioning, data from India on the prevalence of depression and anxiety in psoriasis patients remains limited. This study aimed to evaluate psychological morbidity and its relationship with disease severity and quality of life (QoL) in patients with psoriasis.

Methods

A cross-sectional study was conducted among 63 adult patients with psoriasis attending a tertiary care teaching hospital in Southern India between February 2021 and August 2022. Patients aged 18-65 years with disease duration ≥12 months and without major comorbidities were included. Disease severity was assessed using the Psoriasis Area Severity Index (PASI), QoL was assessed using the Dermatology Life Quality Index (DLQI), and psychological status was assessed using the Depression Anxiety Stress Scale (DASS-21). Statistical analyses included independent t-test, Mann-Whitney U test, chi-square test, and Spearman correlation, with p<0.05 considered significant.

Results

The study population included 42 males (66.7%) and 21 females (33.3%). Female patients were significantly younger than males (34.8±11.9 vs. 44.5±13.1 years, respectively; p=0.006) and had higher DLQI scores (p=0.011). Depression and anxiety were observed in 22.2% and 9.5% of patients, respectively. PASI scores showed a weak positive correlation with DLQI (ρ=0.352; p=0.005). In contrast, DLQI demonstrated a moderate positive correlation with depression, anxiety, and stress scores (ρ=0.540-0.571; p<0.005). Family completion and prior systemic therapy were associated with significantly lower DLQI scores.

Conclusion

QoL correlates more strongly with psychological morbidity than with clinical severity in patients with psoriasis. Psychological burden appears to be only weakly associated with disease severity. Incorporating DLQI into routine dermatology practice may serve as a practical first-step screening tool, with higher scores prompting further evaluation using structured instruments such as DASS-21 and, where indicated, referral for psychiatric assessment.

## Introduction

Psoriasis is a chronic inflammatory disease of the skin characterized by scaly plaques over the body. In some cases, it can also involve the small joints and lead to significant disability. The severity of psoriasis varies over time, and patients often require multiple treatment modalities or prolonged therapy before achieving acceptable control. For many patients, the dermatologist is the first - and sometimes the only - healthcare provider they consult [1].

The prevalence of psoriasis varies widely across populations, ranging from 0.5% to 11.8% globally, with most reported prevalence rates between 0.5% and 2.5% [2-6]. In India, epidemiological data are limited and largely derived from hospital-based studies, which restricts accurate estimation of disease burden [7]. The knowledge gap in psychodermatology data in Indian patients with psoriasis requires attention to improve the quality of care.

However, psoriasis is not merely a disease of the skin. As with any chronic condition, it can significantly affect psychological well-being. The visibility of lesions plays a major role in body image perception, social interactions, and interpersonal relationships. Patients often experience reduced confidence, impaired social functioning, and difficulties in daily life, which may adversely affect treatment adherence and overall quality of life (QoL) [8].

The interaction between the skin and the mind is well recognized in the field of psychodermatology and is mediated through neuroendocrine and immune pathways, collectively described as the neuro-immuno-cutaneous system [9]. Patients with dermatological disorders may develop emotional disturbances, including low self-esteem and distress, and conditions such as psoriasis have been associated with neurotic and dysthymic traits [8,10]. Patients with psoriasis are also at an increased risk of psychiatric comorbidities, including depression, anxiety, and suicidal ideation [11-13]. Despite this, many patients remain undiagnosed, as they may not actively seek psychiatric care.

In routine clinical practice, assessment of psoriasis should extend beyond objective severity. Psoriasis Area Severity Index (PASI) was first introduced in 1978 by Fredriksson and Petersson [14]. While the PASI provides a clinical measure of disease severity, it does not fully capture patient experience. The Dermatology Life Quality Index (DLQI), developed by Finlay and Khan, is a widely used tool that evaluates the impact of skin disease on QoL and has been validated across multiple conditions and populations [15,16]. Similarly, the Depression Anxiety Stress Scale (DASS-21) is a validated instrument used to assess psychological morbidity with good reliability [17]. This tool can simultaneously evaluate depression, anxiety, and stress dimensions and has demonstrated acceptable psychometric validity in people with chronic disease.

Although psoriasis-associated psychological morbidity has been investigated globally, few Indian studies have simultaneously evaluated clinical severity, QoL, and multidimensional psychological morbidity using PASI, DLQI, and DASS-21 together, particularly in Southern India [7]. Therefore, the present study was undertaken to evaluate the prevalence of depression and anxiety in patients with psoriasis and to assess their relationship with disease severity and QoL.

## Materials and methods

Study design and ethical considerations

This was a cross-sectional study conducted among adult patients with psoriasis attending the outpatient department of dermatology at Belagavi Institute of Medical Sciences, Belagavi, India. Patients were also included if they were referred from the outpatient department of psychiatry. The study was carried out over a period of 18 months, from February 2021 to August 2022. Ethical clearance for the study was obtained from the Institutional Ethics Committee of Belagavi Institute of Medical Sciences (Approval No: BIMS-IEC/114/2020-2021) prior to the commencement of the study. The study was conducted in accordance with the principles of the Declaration of Helsinki. The study was prospectively registered in Clinical Trials Registry - India (CTRI/2021/04/032731).

Study participants

Patients between 18 and 65 years of age with a diagnosis of psoriasis for at least 12 months were included in the study. Patients with pre-existing psychiatric, endocrine, cardiovascular, neurological, or rheumatological comorbidities, as well as those with malignancies, were excluded. Informed consent was obtained from all participants prior to inclusion in the study. The sample size was estimated using available epidemiological data because regional prevalence estimates for depression and anxiety among psoriasis patients were limited during protocol development. The estimated prevalence of psoriasis is 2.8% in the adult population [10]; we used it to calculate the minimum required sample size of 42 because of a lack of specific psychodermatological data in the target population. The final sample size exceeded this requirement.

Classification systems

Based on the PASI score, the severity of psoriasis was classified into mild (PASI: 0-4), moderate (PASI: 5-10), and severe (PASI: >10) [14]. DLQI was classified as no effect (score: 0-1), small effect (score: 2-5), moderate effect (score: 6-10), very large effect (score: 11-20), and extremely large effect (score: 21-30) [15]. DASS-21 includes three subscales for depression, anxiety, and stress. For depression, the severity is classified as normal (subscale score: 0-9), mild (subscale score: 10-13), moderate (subscale score: 14-20), severe (subscale score: 21-27), and extremely severe (subscale score: >28). Similarly, the severity of anxiety was classified as normal (subscale score: <8), mild (subscale score: 8-9), moderate (subscale score: 10-14), severe (subscale score: 15-19), and extremely severe (subscale score: >20). Lastly, the severity of stress was classified as normal (subscale score: 0-14), mild (subscale score: 15-18), moderate (subscale score: 19-25), severe (subscale score: 26-33), and extremely severe (subscale score: >34) [17].

Statistical analysis

Data collection was carried out using a structured format at the time of consultation. Baseline demographic details, including age, sex, marital status, education, occupation, and family status, were recorded. Clinical history of psoriasis, including duration of disease and treatment history, was documented. Information regarding current and past therapies was also noted. Disease severity, QoL, and psychological status were assessed using PASI, DLQI, and DASS-21, respectively. Permission to use DLQI was obtained from Cardiff University (license ID: CUQoL4300). The assessments were conducted by the principal investigator, and the questionnaires were interviewer-administered. Data were collected digitally using structured questionnaires administered via Google Forms (Google LLC, Mountain View, CA, USA). Responses were linked to a Google Sheets database (Google LLC), which automatically computed the PASI, DLQI, and DASS-21 subscale scores. These calculations were based on custom JavaScript functions developed by the principal investigator. The calculated results were archived and backed up weekly [18]. The use of this system minimized manual errors and ensured consistency across entries.

The collected data were reviewed and cleaned to remove duplicate and incomplete entries prior to analysis. Statistical analysis was performed using IBM SPSS Statistics for Windows, Version 26 (Released 2018; IBM Corp., Armonk, New York, United States). Continuous variables were tested for normality using the Kolmogorov-Smirnov test. Normally distributed variables were presented as mean ± standard deviation, while non-normally distributed variables were expressed as median with interquartile range. Comparisons between groups were performed using the independent samples t-test for normally distributed variables and the Mann-Whitney U test for non-normally distributed variables. Categorical variables were expressed as frequencies and percentages and compared using the chi-square test. Correlation between continuous variables was assessed using Spearman’s correlation coefficient. Stratified analysis was performed based on age group, sex, marital status, completion of family, and employment status. A p-value of less than 0.05 was considered statistically significant.

## Results

During the study period, 120 patients with psoriasis visited the dermatology outpatient department. Of them, patients were excluded due to the following reasons: age <18 or >65 years (n=7), duration of psoriasis <12 months (n=29), and presence of comorbidities (n=21). Consequently, 63 patients were included in the final analysis. Of these, 42 (66.7%) were males, and 21 (33.3%) were females, with a male-to-female ratio of approximately 2:1 (Table 1). The mean age of the study population was 41.3 ± 13.5 years. Female patients were significantly younger than male patients (34.8±11.9 years vs. 44.5±13.1 years, respectively; p=0.006). A higher proportion of female patients were below 40 years of age compared to males (71.4% vs. 35.7%; p=0.007). There was no significant difference between male and female patients with respect to marital status or completion of family (Table 1). The majority of patients in both groups were married. In terms of education, most patients had completed primary or secondary schooling, with no statistically significant difference between the sexes. Occupational patterns differed, with a majority of female patients being homemakers, while male patients were more commonly employed in private work or self-owned businesses.

**Table 1:** Demographic Characteristics of the Study Population Data are presented as mean±standard deviation or n (%), as appropriate. Comparisons were made between groups using an independent samples t-test.

Variable	Male (n=42)	Female (n=21)	Total (n=63)	p-value
Age (years)	44.5±13.1	34.8±11.9	41.3±13.5	0.006
Age <40 years	15 (35.7%)	15 (71.4%)	30 (47.6%)	0.007
Married	34 (81.0%)	17 (81.0%)	51 (81.0%)	0.228
Completed family	31 (73.8%)	13 (61.9%)	44 (69.8%)	0.332

The median duration of psoriasis in the study population was 60 months, with no significant difference between males and females (Table 2). However, the duration of current treatment was significantly longer in female patients compared to males (six months vs. four months; p=0.005). Most patients were on topical therapy, while a smaller proportion had received systemic treatment such as methotrexate. There was no significant difference in the type of treatment received between male and female patients.

**Table 2:** Clinical Characteristics and Treatment Profile Data are presented as median (interquartile range) or n (%), as appropriate. Groups were compared using the Mann-Whitney U test for independent samples between males and females.

Variable	Male (n=42)	Female (n=21)	Total (n=63)	p-value
Duration of psoriasis (months)	60 (30-180)	84 (36-120)	60 (30-132)	0.781
Duration of current treatment (months)	4 (2-6)	6 (5-8)	5 (3-6)	0.005
Topical therapy	24 (57.1%)	17 (80.6%)	41 (65.1%)	0.062
Systemic therapy (methotrexate)	6 (14.3%)	1 (4.8%)	7 (11.1%)	0.257

Assessment of disease severity, QoL, and psychological status revealed that female patients had significantly higher DLQI scores compared to males (p=0.011), indicating a greater impairment in QoL (Table 3). There was no statistically significant difference in PASI or DASS-21 subscale scores between male and female patients (p>0.05 for all comparisons), despite a significantly higher DLQI score among females (p=0.011). Overall, depression and anxiety were observed in 22.2% and 9.5% of patients, respectively.

**Table 3:** Disease Severity, Quality of Life, and Psychological Outcomes Dermatology Life Quality Index (DLQI) values are presented as median (interquartile range). Prevalence of depression, anxiety, and stress is presented as numbers (percentages). Comparisons were made between male and female patients using an independent samples proportion test.

Variable	Male	Female	Total	p-value
DLQI score	13 (12-15)	17 (13-20)	14 (12-17)	0.011
Depression	10 (23.8%)	4 (19.0%)	14 (22.2%)	0.668
Anxiety	3 (7.1%)	3 (14.3%)	6 (9.5%)	0.363
Stress	0 (0%)	0 (0%)	0 (0%)	0.31

Further analysis showed that DLQI scores varied significantly across age groups (p=0.011), with younger patients demonstrating greater impairment in QoL. In addition, DASS subscale scores showed significant variation across age groups (p=0.049, 0.027, and 0.016 for depression, anxiety, and stress, respectively), indicating a broader age-related influence on psychological morbidity. Patients who had completed their family and those who had received prior systemic therapy had significantly lower DLQI scores (p=0.003 and p<0.001, respectively), suggesting better perceived QoL in these groups (Table 4).

**Table 4:** Group-Wise Comparisons of DLQI and DASS Scores Data are represented by p-values for group-wise comparisons performed using the Kruskal-Wallis test. DLQI: Dermatology Life Quality Index; DASS: Depression Anxiety Stress Scale; PASI: Psoriasis Area Severity Index

Variable	Age Group	Marital Status	Family Completion	Education Level
Duration of psoriasis	0.01	0.188	0.466	0.327
Duration of current treatment	0.373	0.192	0.763	0.971
Duration of past treatment	0.557	0.109	0.427	0.4
PASI score	0.485	0.061	0.117	0.273
DLQI score	0.011	0.208	0.069	0.711
DASS-D score	0.049	0.089	0.44	0.64
DASS-A score	0.027	0.033	0.021	0.367
DASS-S score	0.016	0.004	0.004	0.321

Correlation analysis demonstrated a weak positive correlation between PASI and DLQI scores (Spearman’s ρ=0.352; p=0.005). In contrast, DLQI showed a moderate positive correlation with depression, anxiety, and stress scores (Spearman’s ρ ranging from 0.540 to 0.571; p<0.005), indicating that psychological morbidity was more closely related to QoL than to clinical severity of disease (Table 5).

**Table 5:** Correlation Analysis Correlation between variables was assessed using Spearman’s correlation coefficient, with values expressed as rho (ρ) and p-value. DLQI: Dermatology Life Quality Index; DASS: Depression Anxiety Stress Scale; PASI: Psoriasis Area Severity Index

Variables	Spearman’s rho	p-value
PASI vs. DLQI	0.352	0.005
DLQI vs. DASS-D	0.571	<0.001
DLQI vs. DASS-A	0.54	<0.001
DLQI vs. DASS-S	0.553	<0.001
DASS-D vs. DASS-A	0.628	<0.001
DASS-D vs. DASS-S	0.628	<0.001
DASS-A vs. DASS-S	0.759	<0.001

## Discussion

In this study, we evaluated the psychological burden and QoL in patients with psoriasis and examined their relationship with disease severity. A key finding was that impairment in QoL and psychological morbidity did not parallel clinical severity. While PASI demonstrated only a weak positive correlation with DLQI (ρ=0.352; p=0.005), DLQI showed a moderate positive correlation with depression, anxiety, and stress scores (ρ=0.540-0.571; p<0.005). This suggests that patient-perceived disease burden is more closely aligned with psychological morbidity than with objective clinical severity, highlighting a clinically important disconnect between physician-assessed severity and patient experience. This observation is consistent with previous studies that have demonstrated that subjective measures of disease burden better reflect patient suffering than objective severity indices [19,20]. Furthermore, moderate-to-severe psoriasis has been associated with poorer DLQI scores, reinforcing the importance of incorporating patient-reported outcomes into routine clinical assessment [21].

The prevalence of depression (22.2%) and anxiety (9.5%) observed in our study is comparable to previously reported data. A meta-analysis by Dowlatshahi et al. reported depression in approximately 28% of patients with psoriasis [13]. Similarly, studies have consistently demonstrated that patients with psoriasis have significantly higher odds of anxiety, depression, and suicidal ideation compared to the general population [11-13]. Although we did not observe statistically significant sex differences in DASS-21 scores, the presence of psychological morbidity in a substantial proportion of patients underscores the need for routine screening.

An important finding in our study was that DLQI scores were significantly higher in female patients despite comparable PASI scores, indicating a greater perceived impact of disease on QoL. This suggests that psychosocial factors - such as visibility of lesions, societal expectations, and body image concerns - may disproportionately affect female patients. Previous literature has emphasized that psychosocial burden in dermatological conditions is influenced by individual and sociocultural factors rather than disease severity alone [8].

Age-stratified analysis revealed that younger patients experienced greater impairment in QoL and higher psychological distress. This aligns with earlier observations that psoriasis with onset before 40 years of age is more strongly associated with psychological stress and disease exacerbation [22-24]. Younger individuals may be more affected due to greater social exposure, occupational pressures, and concerns regarding physical appearance.

The role of stress in psoriasis is well-established. Studies have reported that stress precedes disease onset in up to 44% of patients and contributes to exacerbations in up to 80% [20,25]. In addition to psychological factors, biological mechanisms may also contribute to this relationship. Neuropeptides such as substance P and vasoactive intestinal peptide have been implicated in stress-induced exacerbation of psoriatic lesions, suggesting a neuro-immunological basis for disease activity [26].

Another notable observation in our study was that patients who had completed their family and those who had received prior systemic therapy had significantly lower DLQI scores. This may reflect improved disease control in patients receiving systemic therapy. Alternatively, it may indicate psychological adaptation over time, where patients develop coping mechanisms that reduce the perceived burden of disease. Similar behavioral adaptation has been described in chronic dermatological conditions [27]. Patients with psoriasis may also adopt maladaptive coping strategies. Increased prevalence of alcohol consumption and smoking has been reported in this population, both of which are known to worsen disease severity and interfere with treatment outcomes [28-31]. These factors may contribute to a cyclical relationship between poor disease control and psychological distress.

**Figure 1:**
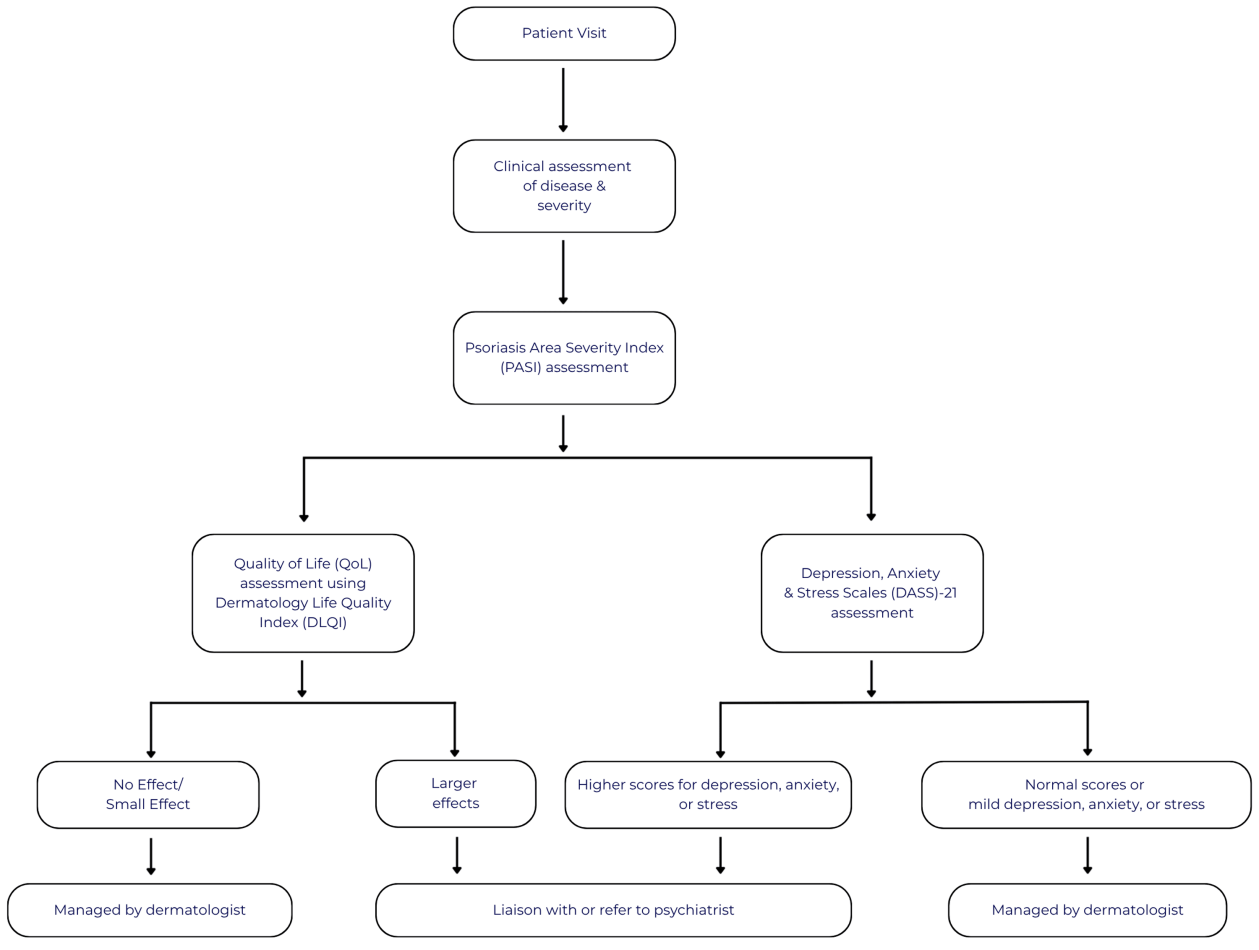
A Proposed Psychodermatology Model of Evaluation and Management of Patients With Psoriasis Patients with no or mild scores on either scale can be managed by dermatologists. However, higher scores warrant liaisoning or referral to a psychiatrist. Image credit: Image created by the authors using Canva (Canva Pty Ltd., Sydney, Australia).

The findings of this study reinforce the concept of psychodermatology, which emphasizes the interaction between psychological processes and dermatological disease through neuroendocrine and immune pathways [22]. Given that dermatologists are often the primary point of care, there is a critical opportunity to identify psychological comorbidities early. From a clinical perspective, our results suggest that DLQI and DASS-21 may serve as practical first-line screening tools in routine dermatology practice. Patients with higher scores on DLQI or DASS-21 can be further managed by dermatologists or referred to psychiatrists for additional management. This integrated approach may improve treatment adherence, patient satisfaction, and overall clinical outcomes (Figure 1).

This study has several limitations. First, it was a hospital-based cross-sectional study at a single centre, which limits the generalizability of our findings and prevents one from deriving any causal inferences. Second, the sample size (n=63), although adequate for exploratory analyses, may be underpowered for detecting smaller effect sizes, especially in subgroup comparisons. Third, self-report tools such as DASS-21 and DLQI, while validated, may be subject to recall bias or social desirability bias. Finally, we excluded patients with known comorbidities, which might limit the extrapolation of our findings because psoriasis frequently coexists with metabolic syndrome and cardiovascular disease.

Future research direction

The findings of this study encourage integrative psychodermatological studies to better understand the healthcare burden in such patients. Additionally, adequate tools must be used to ensure that precise information about dermatological diseases, as well as mental health, is captured. Lastly, the field of psychodermatology is in its nascent stages of large-scale studies. Therefore, researchers are encouraged to explore population studies for better insights and outcomes for these patients.

## Conclusions

In this hospital-based cross-sectional study in patients with psoriasis, a majority of patients had mild-to-moderate psoriasis; however, the effect of psoriasis on their QoL was notably high, with DLQI scores indicating a very large effect on daily functioning. Depression and anxiety were prevalent in 22.2% and 9.5% of the cohort, respectively. Younger age and female sex were significantly associated with higher DLQI and DASS scores. In contrast, being married, having completed one’s family, and a history of systemic therapy were associated with better QoL and lower levels of psychological distress. Our findings highlight the need for a holistic and patient-centered approach to psoriasis management - one that integrates psychosocial screening and support alongside clinical care, particularly in younger and female patients.
